# Effect of dietary gossypol supplement on fermentation characteristics and bacterial diversity in the rumen of sheep

**DOI:** 10.1371/journal.pone.0234378

**Published:** 2020-06-10

**Authors:** CaiDie Wang, YuQi Li, TunNiSa MaiTiSaiYiDi, HongJian Yang, KaiLun Yang

**Affiliations:** 1 Xinjiang Key Laboratory of Meat and Milk Production Herbivore Nutrition, College of Animal Science, Xinjiang Agricultural University, Urumqi, Xinjiang, The People’s Republic of China; 2 State Key Laboratory of Animal Nutrition, College of Animal Science and Technology, China Agricultural University, Beijing, The People’s Republic of China; Hubei University, CHINA

## Abstract

The tolerance of ruminants to gossypol, a natural phenolic compound derived from the cotton plant, is greater than that of monogastric animals, partially because of the gossypol-degrading bacteria in the rumen of the ruminants. In this study, we aimed to examine the effect of gossypol supplementation on fermentation characteristics, bacterial α**-**diversity and community structure in the rumen fluid of sheep to analyse the change of bacterial in response to gossypol. 8 sheep with permanent fistula were randomly divided into 2 groups, a control and gossypol acetate supplementation groups. Sheep in the latter group were supplemented with gossypol acetate at the levels of 600 mg and 1,200 mg/animal per day during the first (S1, days 1 to 27) and subsequent (S2, days 28 to 47) stages. Gossypol supplementation significantly increased the molar proportion of acetate, and decreased the molar proportion of isobutyric acid, butyric acid, and isovaleric acid in the rumen fluid. Gossypol supplementation have no significant effect on bacterial diversity in the rumen fluid. At the phylum level, gossypol had no effect on bacterial community. At the genus level, gossypol supplementation significantly increased the relative abundance of *Treponema_2*. However, there were no significant differences in the relative abundance of dominant bacterial genera. In conclusion, gossypol supplementation had an effect on molar proportion of acetate, isobutyric acid, butyric acid, and isovaleric acid, but had no significant effect on the bacterial diversity and relative abundance of dominant bacteria in rumen fluid of sheep.

## Introduction

Whole cottonseeds are rich in protein [1], energy [2], and fibre [3] and are extenstively used as an important feed ingredient, especially for high-yield dairy cows. However, the presence of gossypol hinders the potential use of cottonseed by-products in farm animal feeding. Gossypol is a toxic phenolic compound derived from the cotton plant, with the greatest concentration found in cottonseeds [4]. The presence of gossypol can enhance the resistance to pests for genus *Gossypium* [5]. Numerous *in vitro* studies have shown that gossypol has anticancer [6], antivirus [7], antimicrobial [8], and antiparasitic [9–10] properties. For a long time, it was believed that ruminants in comparison with monogastric animals are believed to be more tolerant to free gossypol [11]. Currently, two possible mechanisms have been proposed to explain gossypol detoxification in ruminant animals. One explanation is that the free gossypol can be converted into bound gossypol by binding to soluble proteins [12]. The other is that gossypol can be used as a carbon source by rumen microorganisms, assuming that gossypol is degraded into nontoxic metabolites [13].

Bacteria (10^10^ to 10^11^ CFU/mL) are the most abundant microorganisms in the rumen ecosystem compared with protozoa, fungi, and methanogens [14]. Previous studies have mainly addressed the feeding effects of whole cottonseeds on dry matter intake (DMI) in dairy cows [15]. In consecutive batch cultures, gossypol initially causes a decrease in the level of rumen microorganisms at first exposure, however, these microorganisms are able to adapt following prolonged exposure to gossypol [16]. Although the application of high-throughput sequencing technologies has increased the ability to study microbial communities at a high taxonomic resolution [17–18], limited information is available regarding the effect of gossypol on rumen bacterial diversity. It is also not clear if gossypol dietary intake could change the dominant bacteria composition in the rumen. In the present study, high-throughput sequencing technology was applied in a sheep feeding trial, and the main objective was to determine if dietary gossypol supplementation could shift rumen fermentation and bacteria composition in rumen fluid.

## Material and methods

The experimental protocol was approved (animal protocol number: 2017006) by the Animal Care and Use Committee of Xinjiang Agricultural University, Urumqi, Xinjiang, China. After the experiment, the sheep were housed for use in further research. The study was conducted from August to October 2017 at Hui Kang Animal Husbandry Biotechnology Co., Ltd. breeding farm in Urumqi, Xinjiang, China.

### Animals, diet, and experimental procedure

Eight healthy 3-year-old female adult Kazakh sheep with an average body weight of 49.13 ± 4.70 kg were served as experimental animal, and each animal was surgically fitted with a rumen fistula (2 cm diameter). The animals were randomly arranged into 2 groups with 4 sheep per group. The sheep had free access to water and were individually kept in separate cages and fed the same basal diet (Table 1) twice daily at 08:00 and 20:00 for 47 days. During feeding trial, sheep in the control group were fed the basal diet, and sheep in the gossypol group were fed a basal diet supplemented with gossypol acetate (98% purity, Hubei Xin Yuan Shun chemical Co., Ltd., Hubei province, China). Dietary gossypol acetate levels in the gossypol group were 600 mg and 1,200 mg/animal per day during the first stage (S1, days 1 to 27) and the subsequent stage (S2, days 28 to 47). The gossypol choice of 1200 mg/animal in the present study was set based on the maximum limit allowance when in ruminant feeds (cotton products) in China [19]. To facilitate the gossypol administration, sheep in the gossypol treatment group were individually offered the experimental amounts of gossypol acetate after being mixed with 50 g of a powdered commercial concentrate (Tian Kang Animal Husbandry Biotechnology Co., Ltd., Xinjiang, China) in advance, whereas the sheep in the control group was fed only with the 50 g of the same commercial concentrate. Ingredients and nutritional levels of the commercial concentrate are listed in S1 Table. Afterwards, the same basal diet in Table 1 were provided ad libitum. Initial live body weight and final live body weight were weighed and recorded to calculated average daily gain (ADG), and dry matter intake of each sheep was measured according to difference of the diet offered and leftovers in the troughs throughout the whole feeding trial.

**Table 1:** Feed ingredients and chemical composition of the sheep diet (DM basis).

Ingredient (% as fed basis)	Content	Nutrient level (% as fed basis)	Content
Grass silage	12.64	NDF	50.41
Alfalfa hay	25.05	ADF	29.84
Wheat Straw	25.67	Crude protein	12.55
Corn meal	16.12	Calcium	1.01
Oat meal	5.86	Phosphorus	0.28
Barley meal	5.5		
Soybean meal	7.32		
CaHPO_4_	1.1		
NaCl	0.37		
Premix^1)^	0.37		
Total	100		

NDF, Neutral detergent fibre; ADF, Acid detergent fibre

^1)^ The premix provided the following per kilogram of diet: vitamin A 480 IU, vitamin B_1_ 816 mg, vitamin B_2_ 333 mg, vitamin B_6_ 49 mg, vitamin D 70 U, vitamin E 21333 IU, pantothenic acid 20 mg, nicotinamide 485 mg, Cu (as copper sulphate) 11 mg, Fe (as ferrous sulphate) 35 mg, Mn (as manganese sulphate) 33 mg, Zn (as zinc sulphate) 31 mg, I (as potassium iodide) 2 mg, Se (as sodium selenite) 6 mg, and Co (as cobalt chloride) 1 mg.

### Sample collection

During the 47-day feeding trial, rumen contents were collected 3 h after the morning feeding on day 5, 10, 15, 20 of S1 stage and day 32, 37, 42 and 47 of S2 stage and were filtered using a nylon bag (pore size of 250 *μ*m). The filtrated rumen fluid (20 mL) of each sheep was sampled and sub-packed into sterilized cryopreservation tubes, immediately frozen in liquid nitrogen, and then stored at −80°C. All rumen fluid samples were used for ammonia N (NH_3_-N) and volatile fatty acid (VFA) analysis except that the samples collected on day 20 and day 47 were used for bacterial DNA extraction and sequencing.

### Rumen NH_3_-N and VFAs measurement

An indophenol colorimetric reaction was used to determine the ammonia N concentration in rumen fluid. phenolic absorbance at 630 nm was measured using a spectrophotometer [20]. Gas chromatography (GC-2010, Shimadzu, Japan) was used to measure the VFA concentration in the sheep rumen fluid [21]. And 4-methyl valaric was used as internal standard. The following chromatographic conditions were used: capillary column 30 m × 0.32 mm × 0.25 mm film thickness, column temperature 150°C, and detector temperature 220°C.

### DNA extraction and sequencing

The thawed samples of rumen fluid (1.5 mL) were centrifuged at 800 g for 5 min at 4°C, then the supernatant was centrifuged at 10,000 g for 10 min at 4°C. The sediments of the second centrifuged samples were used to extracted the total DNA. Total genomic DNA was extracted using the CTAB/SDS method, the procedure was operated in a clean bench. The concentration and purity of the total genome DNA were assessed following a separation on 0.8% agarose gels. The DNA was diluted to 1 ng/*μ*L using sterile water before analysis. The bacterial 16S rDNA V3-V4 primers were 341F: 5′-CCTAYGGGRBGCASCAG-3′ and 806R: 5′-GGACTACNNGGGTATCTAAT-3′. All polymerase chain reactions (PCRs) were conducted using a Phusion® High-Fidelity PCR Master Mix. Briefly, PCR was in a 25 *μ*L reaction mixture containing 10 ng of DNA, 0.2 *μ*M of each primer, 12.5 *μ*L of Phusion® High-Fidelity PCR Master Mix. The PCR program was as follows: 95°C for 30 s, followed by 30 cycles of 95°C for 10 s, 55°C for 30 s, 72°C for 30 s, and last 72°C for 5 min. The TruSeq® DNA PCR-Free Sample Preparation Kit (Illumina, San Diego, California, USA) was used to generate the sequencing libraries. Library qualities were evaluated using a Qubit 2.0 Fluorometer (Thermo Scientific, Massachusetts, USA) and the Agilent Bioanalyzer 2100 System. Finally, the IonS5^TM^ XL platform was used to sequence the libraries, generating 600-bp single-end reads. The DNA extraction, PCR, and sequencing were completed by Beijing Novogene biology Co., Ltd.

### Sequence data processing

Cutadapt version 1.9.1 [22] was used to remove low-quality (quality values of less than 17) parts of the reads. Using the barcode, each sample data were obtained from the reads. The truncated barcode and primer sequences were used for the initial quality control to obtain the raw reads. The UCHIME algorithm [23] was used to compare the raw read sequences to the database to detect chimeric sequences [24] and then to remove them to obtain clean data. Raw data were available at the NCBI Sequence Read Archive, BioProject accession number: PRJNA597568.

Uparse version 7.0.1001 [25] was used to cluster the clean data sequences into operational taxonomic units (OTUs) with 97% identity. Using the Mothur method and SILVA 128 database [26,27] to perform species annotation analysis (the threshold was set from 0.8 to 1). Finally, the sample data were normalized using R-2.15.3, and normalization was performed based on the smallest amount of data in the sample (Random sampling: equal proportion of each OTU were selected). To removing chloroplast, mitochondrial, archaeal, eukaryotic and unidentified reads. QIIME (version 1.9.1) was used to calculate the α-diversity of bacterial community in the rumen fluid of sheep. Simpson and Shannon are usually used to estimate the community diversity. However, regarding diversity, not only the qualitative amount of species, but also the abundance of the species must be taken into account. Chao1 and ACE are used to estimate the community richness. The Goods coverage is used to estimate the sequencing depth. R 3.5.1 were used to perform non-metric multidimensional scaling (NMDS) analysis. To further examine the effect of gossypol on the pattern of the bacterial in rumen fluid, the permutational multivariate analysis of variance using distance matrices (PERMANOVA) based on Binary Jaccard dissimilarity matrix were conducted.

### Statistical analysis

Data were analysed using the MIXED procedure of the SAS software (version 9.2; SAS Institute, Cary, NC, USA). The model was applied as follow:

$$Y_{ijk}=\mu +T_{i}+S_{j}+{\left(T\times S\right)}_{ij}+C_{k}+e_{ijk}$$

Where, *Y*_*ijk*_ was the response variable, *μ* is the overall mean, *T*_*i*_ was the fixed effect of the treatment (control vs gossypol), *Sj* was the fixed effect of stage (j = S1 and S2), *C*_*k*_ was the random effect of the animal (k = 1 to 6) and e_*ijk*_ was the residual error. First-order utoregressive and compound symmetry (homogenous and heterogeneous) were tested as covariance structures, and the covariance structure with the lowest Akaike’s Information Criterion was retained in the final model. The least square means and standard errors were estimated with the LSMEANS statement of the SAS software (version 9.2; SAS Institute, Cary, NC, USA). The means between the control and gossypol administration were compared with Tukey’s method. Significance was declared at *P* < 0.05 unless otherwise noted.

## Results

### Live body weight and DMI response to dietary gossypol supplement

As shown in Table 2, initial body weight of sheep did not differ between the control and gossypol group. No difference for final body weight was observed between two groups though ADG was numerically increased in gossypol group in comparison with the control. Increasing gossypol feeding up to 1,200 mg/animal numerically decreased ADMI by 4%. Consequently, feed: gain ratio was slightly decreased in gossypol group compared with the control though such decrease was not statistically significant.

**Table 2:** Effect of dietary gossypol supplement on body weight change and feed intake of adult female sheep.

	Treatment^2^			*P*-value^3^		
Items^1^	Control	Gossypol	SEM	G	S	G × S
Initial LBW, kg	49.4	48.7	2.53	0.85	-	-
Final LBW, kg	56.5	55.9	2.42	0.87	-	-
ADG, g/d	178	196	22.3	0.45	-	-
ADMI, kg/d						
Day 1–27	1.67	1.67	0.082	0.77	0.11	<0.01
Day 28–47	1.69	1.62				
Day 1–47	1.68	1.65	0.013	0.11	-	-
Feed: Gain ratio	10.3	9.9	1.56	0.73	-	-

^1^ LBW: Live body weight, ADG: Average daily gain, ADMI: Average dry matter intake, SEM: Standard error of the least square means.

^2^ Daily dosage in the gossypol group were 600 and 1,200 mg per animal during the first stage (days 1–27) and subsequent stage (days 28–47). The data is for least square means.

^3^ G: Effect of gossypol supplement, S: Effect of feeding stage, G×S: Interaction effect between gossypol treatment and feeding stage.

### Gossypol supplementation effects on fermentation parameters

As shown in Table 3, Compared with the control group, gossypol significantly decreased the molar proportion of isovaleric acid during S1. Regarding the effect of gossypol intake levels (600 mg vs 1,200 mg), supplementation with gossypol acetate significantly increased the molar proportion of acetate (*P* < 0.01), and significantly decreased the molar proportion of isobutyric acid (*P* = 0.02), butyric acid (*P* < 0.01), and isovaleric acid (*P* = 0.01) in the sheep rumen fluid, but had no significant effect on the concentration of NH_3_-N, tVFA and the molar proportion of propionate, and valeric acid.

**Table 3:** Effect of gossypol supplementation on the rumen fermentation characteristics of adult sheep.

Items	Stage^1^	Treatment^2^		SEM	*P*-value^3^		
		Control	Gossypol		G	S	G × S
NH_3_-N, mg/dL	S1	39.5	33.2	3.11	0.42	0.45	0.06
	S2	35.7	34.9				
tVFA, mmol/L	S1	82.6	83.1	1.16	0.20	0.28	0.44
	S2	80.3	82.7				
Acetate, %	S1	67.3	67.9	0.19	<0.01	<0.01	0.44
	S2	68.5	68.8				
Propionate, %	S1	17.59	17.47	0.131	0.99	0.16	0.45
	S2	17.23	17.35				
Isobutyric acid, %	S1	0.26	0.22	0.010	0.02	<0.01	0.46
	S2	0.18	0.16				
Butyric acid, %	S1	12.96	12.64	0.088	<0.01	<0.01	0.89
	S2	12.23	11.94				
Isovaleric acid, %	S1	0.65^a^	0.58^b^	0.01	0.01	0.01	0.13
	S2	0.60	0.57				
Valeric acid, %	S1	1.27	1.21	0.027	0.12	0.59	0.46
	S2	1.23	1.21				

^1^ Dietary gossypol acetate inclusions in the gossypol group were 600 and 1,200 mg per animal during the first stage (S1, days 1–27) and subsequent stage (S2, days 28–47).

^2^ The data is for least square means.

^3^ G: Effect of gossypol supplement, S: Effect of feeding stage, G×S: Interaction effect between gossypol treatment and feeding stage.

tVFA, Total volatile fatty acid. SEM: Standard error of the least square means.

^ab^ Values in the same row with different superscripts are significantly different (*p* < 0.05).

### Taxonomic characterization of microbiota in the rumen fluid of sheep

**Fig 1:**
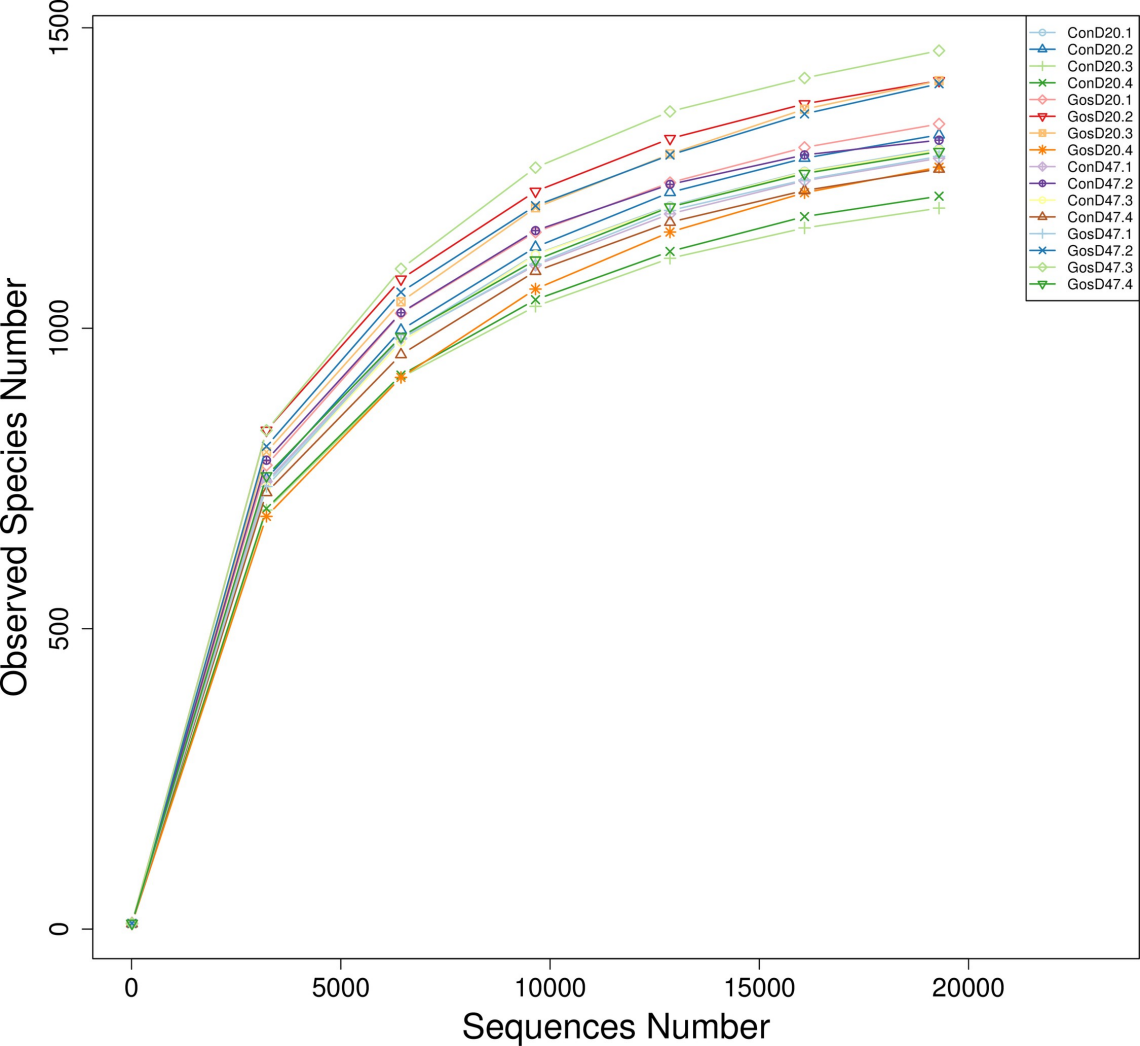
Rarefaction curve of bacteria in the rumen fluid of sheep.

A total of 851,105 clean reads were detected by high-throughput sequencing in 16 samples, with an average of 53,194 reads per sample (S2 Table). The clean reads were annotated into 4,189 OTUs, belonging to 19 phylum, 37 classes, 61 orders, 86 families and 185 genera. In Fig 1, the rarefaction curve tends to be flat, indicating that only a few new species appeared when the sequencing depth was increased, the sequencing depth covered most of the bacteria in the rumen fluid.

### Gossypol supplementation effects on the bacteria α-diversity in rumen fluid

As shown in Table 4, regarding the effect of the gossypol intake level, supplementation with gossypol acetate, the diversity indexes of OTUs (*P* = 0.009), Chao1 (*P* = 0.09) and ACE (*P* = 0.08) have a trend increase. While there were no difference on Shannon and Simpson indexes. The effect of the gossypol on goods coverage of bacterial in the rumen fluid were approximately 1 for the two groups, indicating that the sequencing depth of each sample satisfied the condition for subsequent analysis.

**Table 4:** Effect of gossypol supplementation on the bacterial α-diversity in the rumen fluid of adult sheep.

Items	Stage^1^	Treatment^2^		SEM	*P*-value^3^		
		Control	Gossypol		G	S	G × S
OTUs	S1	1189	1283	30	0.09	0.29	0.39
	S2	1229	1287				
Shannon	S1	8.05	8.35	0.165	0.12	0.27	0.97
	S2	8.26	8.55				
Simpson	S1	0.97	0.98	0.005	0.15	0.49	0.89
	S2	0.98	0.98				
Chao1	S1	1197	1295	30.8	0.09	0.26	0.37
	S2	1239	1300				
ACE	S1	1214	1315	30.9	0.08	0.27	0.35
	S2	1255	1319				
Goods coverage^4^	S1	0.9967	0.9958	0.0004	0.07	0.91	0.91
	S2	0.9967	0.9959				

^1^ Dietary gossypol acetate inclusions in the gossypol group were 600 and 1,200 mg per animal during the first stage (S1, days 1–27) and subsequent stage (S2, days 28–47).

^2^ The data is for least square means.

^3^ G: Effect of gossypol supplement, S: Effect of feeding stage, G×S: Interaction effect between gossypol treatment and feeding stage.

^4^ Represents the sequencing coverage for each sample.

OTUs, Operation taxonomy units. SEM: Standard error of the least square means.

### Analyses of bacterial community structure

**Fig 2:**
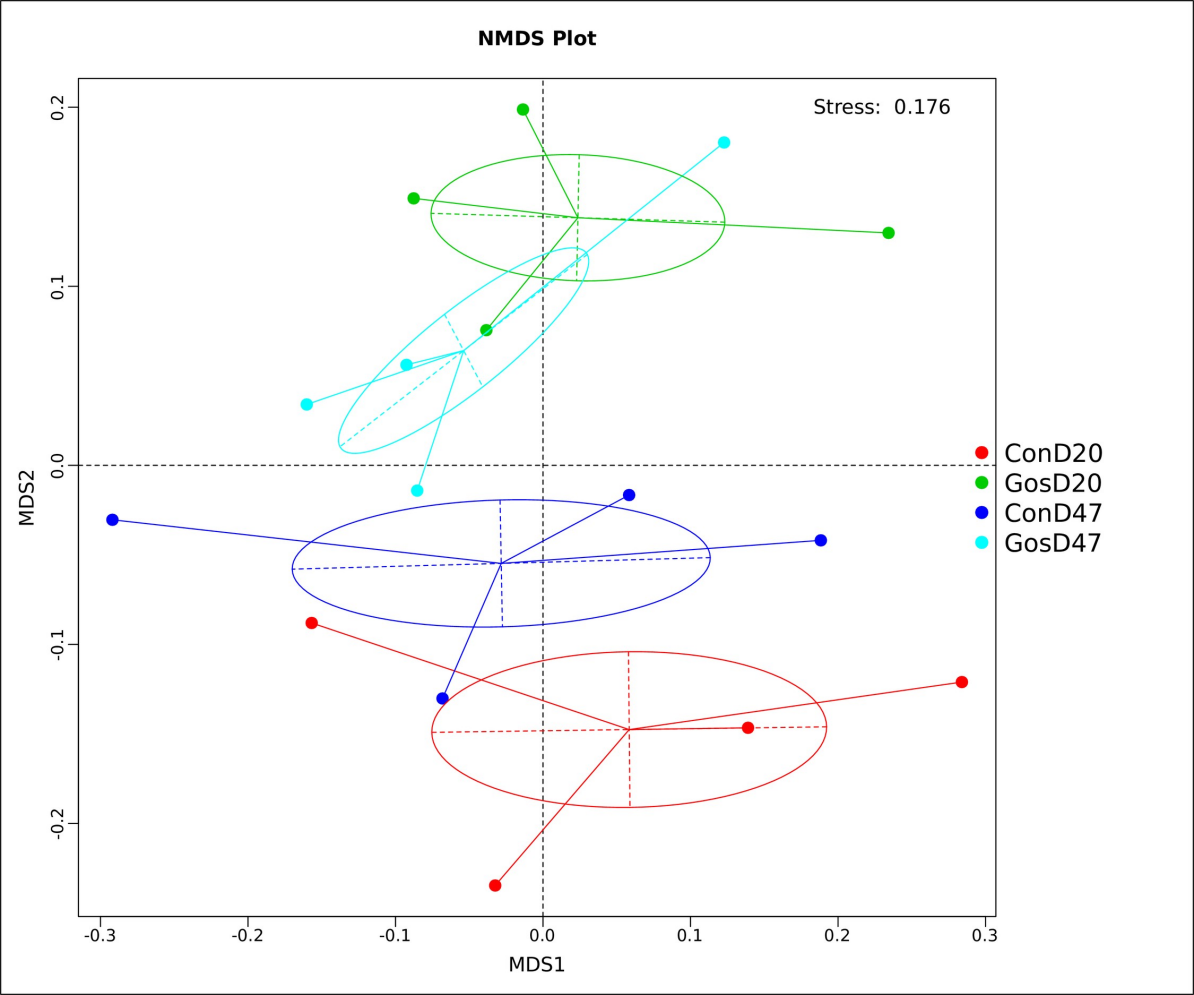
Non-metric multidimensional scaling (NMDS) plot of Binary Jaccard distance between rumen bacterial communities.

The NMDS plot (Fig 2) of bacterial in the rumen fluid were clustered into 4 groups, with the samples in the gossypol group separated from those in the control group obviously at day 20 (S1), showed a shift in the microbial communities of the rumen fluid in sheep by supplementation with gossypol. The bacterial community trends tend towards closeness, between groups, at day 47 (S2). Results of the testing indicated no statistically significant differences in bacterial community between gossypol and control group at day 20 (F = 1.22, R squared = 0.16, *P* = 0.15), and at day 47 (F = 0.98, R squared = 0.14, *P* = 0.53) based on PERMANOVA analyse. The difference of results between NMDS and PERMANOVA anlysis, may be due to different analysis methods and sample size.

### Gossypol supplementation effects on the relative abundance of bacteria at the phylum level in rumen fluid

At the phylum level (Table 5), the dominant bacteria in the sheep rumen fluid were *Bacteroidetes* and *Firmicutes*. Gossypol supplementation had little impact on the relative abundance of the major phyla. Compared with the control, there were no significant differences in the relative abundance of phylum level bacteria at S1 and S2. Regarding the effect of gossypol intake levels (600 mg vs 1,200 mg), there were no significant differences in the relative abundance of phylum-level bacteria (*P* > 0.05). However, the supplementation time significantly affected the relative abundance of *Tenericutes* (*P* = 0.01). After supplement with gossypol acetate, the relative abundance of *Spirochaetes* has a trend to decrease (*P* = 0.06).

**Table 5:** Effect of gossypol supplementation on the relative abundance of bacteria at the phylum level (> 1%) in the rumen fluid of adult sheep [%].

Phylum	Stage^1^	Treatment^2^		SEM	*P*-value^3^		
		Control	Gossypol		G	S	G × S
*Bacteroidetes*	S1	54.6	52.0	2.79	0.61	0.36	0.71
	S2	51.9	50.9				
*Firmicutes*	S1	26.3	30.5	2.02	0.58	0.73	0.16
	S2	28.5	26.9				
*Proteobacteria*	S1	5.7	6.7	1.14	0.41	0.39	0.77
	S2	6.2	7.7				
*Cyanobacteria*	S1	4.35	4.16	0.635	0.41	0.12	0.27
	S2	4.75	6.07				
*Fibrobacteres*	S1	4.0	2.5	0.70	0.73	0.63	0.08
	S2	2.5	3.4				
*Spirochaetes*	S1	1.83	1.24	0.261	0.06	0.19	0.51
	S2	2.24	1.38				
*Tenericutes*	S1	1.32	1.20	0.171	0.68	0.01	0.80
	S2	1.59	1.51				

^1^ Dietary gossypol acetate inclusions in the gossypol group were 600 and 1,200 mg per animal during the first stage (S1, days 1–27) and subsequent stage (S2, days 28–47).

^2^ The data is for least square means.

^3^ G: effect of gossypol supplement, S: effect of feeding stage, G×S: interaction effect between gossypol treatment and feeding stage.

SEM: Standard error of the least square means.

### Gossypol supplementation effects on the relative abundance of bacteria at the genus level in rumen fluid

At the genus level (Table 6), the dominant bacteria in the sheep rumen fluid was *Prevotella_1*. Gossypol supplementation had little impact on the relative abundance of the major genera. Gossypol treatment significantly decreased the relative abundance of *Treponema_2* (*P* = 0.03). After supplement with gossypol acetate, the relative abundance of *Ruminobacter* (*P* = 0.07) have a trend to significant difference. All other genus levels of liquid-phase bacteria were not affected by gossypol treatment.

**Table 6:** Effect of gossypol supplementation on the relative abundance of bacteria at the genus level (> 1%) in the rumen fluid of adult sheep [%].

Genus	Stage^1^	Treatment^2^		SEM	*P*-value^3^		
		Control	Gossypol		G	S	G × S
*Prevotella_1*	S1	15.1	16.7	1.67	0.56	0.08	0.77
	S2	17.9	18.8				
*Rikenellaceae_RC9*	S1	10.2	11.0	1.43	0.71	0.07	0.89
	S2	7.8	8.3				
*Succinivibrionaceae_UCG-002*	S1	1.7	3.3	0.80	0.13	0.83	0.87
	S2	2.0	3.3				
*Prevotellaceae_UCG-003*	S1	3.7	4.4	0.67	0.79	0.45	0.33
	S2	3.9	2.9				
*Fibrobacter*	S1	4.0	2.5	0.70	0.73	0.63	0.08
	S2	2.5	3.4				
*Christensenellaceae_R-7*	S1	3.0	4.8	0.58	0.46	0.24	0.04
	S2	3.7	2.9				
*Ruminococcaceae_NK4A214*	S1	2.42	3.86	0.439	0.14	0.25	0.17
	S2	2.54	2.67				
*Erysipelotrichaceae_UCG-004*	S1	2.10	2.08	0.594	0.95	<0.01	0.62
	S2	2.66	2.79				
*Prevotellaceae_UCG-001*	S1	1.28	1.50	0.417	0.25	0.04	0.16
	S2	1.56	2.65				
*Ruminococcaceae_UCG-002*	S1	2.06	1.87	0.315	0.61	0.27	0.93
	S2	1.61	1.49				
*Ruminococcus_2*	S1	0.35	1.01	0.317	0.40	0.40	0.29
	S2	0.44	0.33				
*Ruminococcaceae_UCG-014*	S1	1.55	1.84	0.271	0.38	0.94	0.84
	S2	1.59	1.75				
*Ruminobacter*	S1	0.67	0.95	0.210	0.07	0.29	0.31
	S2	0.68	1.39				
*Ruminococcaceae_UCG-005*	S1	0.57	0.67	0.225	0.49	012	0.55
	S2	0.77	1.07				
*Ruminococcaceae_UCG-010*	S1	1.20	1.18	0.172	0.82	0.49	0.94
	S2	1.32	1.26				
*Treponema_2*	S1	0.87	0.52	0.156	0.03	0.05	0.78
	S2	1.33	0.88				
*Sphaerochaeta*	S1	1.11	0.54	0.173	0.24	0.052	<0.01
	S2	0.68	0.64				
*Coprostanoligenes*	S1	0.9	1.0	0.14	0.41	0.79	0.42
	S2	1.0	0.8				
*Succiniclasticum*	S1	0.84	1.11	0.210	0.69	0.19	0.26
	S2	0.81	0.78				

^1^ Dietary gossypol acetate inclusions in the gossypol group were 600 and 1,200 mg per animal during the first stage (S1, days 1–27) and subsequent stage (S2, days 28–47).

^2^ The data is for least square means.

^3^ G: Effect of gossypol supplement, S: Effect of feeding stage, G×S: Interaction effect between gossypol treatment and feeding stage.

SEM: Standard error of the least square means.

## Discussion

Gossypol as an anti-nutritional factor hinders the use of cotton by-products in animal diets. In our present study, the DMI of sheep, rumen fermentation characteristics and bacterial diversity in rumen fluid were reported after supplementation with gossypol. The result showed that gossypol supplementation significantly increased the molar proportion of acetate, and significantly decreased the molar proportion of isobutyric acid, butyric acid, and isovaleric acid in the sheep rumen fluid. But supplemented with gossypol had no significant effect on DMI and on the relative abundance of dominant bacterial phylum and genus.

In our study, we found that gossypol supplementation had no impact on DMI. This result is similar to previous reports showing that different feed levels of gossypol contained in cottonseed had no impact on DMI in sheep [28]. Similarly, no difference in feed consumption was found when comparing high and low levels of gossypol feed in lambs [29]. These data suggest that the total nutrient intake in control and gossypol sheep groups was similar.

Ismartoyo found that the concentration of acetate, propionate, and butyric acid were changed by supplementation with the highest level of whole cottonseed in sheep [30]. Previous studies used cottonseed, which also contains other anti-nutritional factors that may have effect on rumen microbiome, such as tannins, clopropenoid fatty acids, etc. However, our study used gossypol acetate as the research object. In our study, supplementation with gossypol acetate significantly effected the proportion of acetate, butyric acid, isovaleric acid and valeric acid in sheep rumen fluid. The effect of gossypol on rumen fluid fermentation characteristics was well studied.

In this study we used next-generation sequencing technology to provide a direct estimate of the effect of gossypol on the bacterial community in the sheep rumen. The high-throughput sequencing analysis showed that, after the appropriate quality control, there were 4,189 OTUs (1,247 OTUs per sample). After supplemented with gossypol acetate, the diversity indexes of OTUs, Chao1 and ACE have a trend increase. It has been shown that gossypol decreases the number of rumen microorganisms at first exposure, but that the number of microorganisms recovers after prolonged gossypol exposure [15]. Previous studies have studied the effect of gossypol on the number of rumen microorganisms. Similarly, studies have shown that rumen microorganisms can degrade or tolerate allyl cyanide and other substances and quickly adapt to these toxic substances [31]. It has also been shown that oxalate significantly increases the relative abundance of bacteria capable of oxalate degradation in mammalian herbivores [32] The diversity of rumen microorganisms is a benefit for balancing the rumen environment [33].

For the NMDS plot reviewed that control and gossypol treatment tended to converge in microbial community at day 47 (S2) relative to day 20 (S1). It may be due to the long-term addition of gossypol, the rumen microorganisms became adaptive to gossypol. This was consistent with that microorganisms are able to adapt following prolonged exposure to gossypol [16].

At the phylum level, *Bacteroidetes* and *Firmicutes* were found to be the dominant phyla in the sheep rumen fluid. this finding is consistent with findings in lamb [34] and calves [35]. Here, we found that compared to sheep in the control group, the relative abundance of *Spirochaetes* in the rumen was found to trend deceased following supplementation with gossypol. Studies have suggested that *Spirochaetes* are associated with diarrhoea in the intestine [36,37]. It is suggested that we need to pay attention to the intestinal health when using cotton by-products containing gossypol as feed material. However, it is not clear why the levels of these bacteria change following supplementation with gossypol. Future studies should evaluate differences in function and metabolism in the rumen after supplementation with gossypol. Gossypol treatment had no effect on the relative abundance of bacterial at phylum level.

At the genus level, *Prevotella_1* was found to be the dominant bacteria in the rumen of sheep, which is consistent with previous studies [38]. Supplementation with gossypol acetate decreased the relative abundance of *Treponema_2* and the relative abundance of *Ruminobacter* have a trend to significant difference. *Ruminobacter* ferment products including succinate, acetate and formate [39], which is consistent with the increase in acetate concentration seen following supplementation with gossypol acetate.

Gossypol has been shown to have a strong inhibitory effect on gram-positive bacteria than on gram-negative bacteria. For example, in one study, 100 *μ*g/mL gossypol could inhibit all gram-positive bacteria, but only 16 of the 45 gram-negative strains used were inhibited at a concentration of 200 *μ*g/mL [40]. It has been reported that 3 *μ*g/mL gossypol can inhibit the growth of *Edwardsiella ictaluri*, but that a sterilization effect was still not achieved at 100 *μ*g/mL [8]. The minimum inhibitory concentration of gossypol has been reported to be 10 *μ*g/mL for *Staphylococcus aureus* and *Leuconostoc mesenteroides*, 25 *μ*g/mL for *Sarcina lutea* and *Bacillus licheniformis*, and 50 *μ*g/mL for *Bacillus polymyxa*, *Bacillus megaterium*, *Bacillus cereus*, and *Bacillus thermoacidurans* [41]. In our previous study, it was found that the concentration of gossypol in the rumen liquid was between 1.50 *μ*g/mL and 2.36 *μ*g/mL [42]. Therefore, the concentration of gossypol in rumen fluid was obviously decreased. Plant secondary metabolites will accumulate when the rate of ingestion exceeds the rate of detoxification [43]. The concentration of gossypol in the rumen fluid did not reach the minimum inhibitory concentration for major bacteria in the rumen fluid. Therefore, undigested gossypol will accumulate in the rumen fluid. Our results revealed that gossypol acetate affected low abundance rumen bacteria, but there was no significant effect on the dominant bacteria. This partly explained the reason that ruminants are tolerant to gossypol, and provide guidance for the application of cotton by-products in animal feed.

## Conclusions

In summary, the levels of gossypol intake used here did not affect feed intake of sheep. Supplementation with gossypol acetate significantly increased the molar proportion of acetate, and significantly decreased the molar proportion of isobutyric acid, butyric acid, and isovaleric acid in the sheep rumen fluid. However, gossypol supplementation had no significant effect on bacteria diversity and the relative abundance of major phylum and genus.

## Supporting information

S1 Table Powder concentrate and nutrition levels of the diet (DM basis). (DOC)

S2 Table Information about the number of reads per sheep per sampling time. (DOC)
