# Supplementary material for: Effect of dietary gossypol supplement on fermentation characteristics and bacterial diversity in the rumen of sheep
Source: PLoS One. 2020 Jun 10;15(6):e0234378. doi: 10.1371/journal.pone.0234378 (PMC7286523; doi:10.1371/journal.pone.0234378)
Supplement: S1 Table — (DOC) [file pone.0234378.s001.doc]

**S1 Table. Powder concentrate and nutrition levels of the diet (DM basis）**

| [**Formulation**](http://dict.cnki.net/dict_result.aspx?searchword=配方&tjType=sentence&style=&t=formulation) **of powder concentrate** | | **Nutrient levels** | |
| --- | --- | --- | --- |
| [**Material**](http://dict.cnki.net/dict_result.aspx?searchword=原料&tjType=sentence&style=&t=material) **name** | **Content of** [**material**](http://dict.cnki.net/dict_result.aspx?searchword=原料&tjType=sentence&style=&t=material)**/%** | **Nutrient name** | **Content/%** |
| Corn | 44.00 | DM | 91.28 |
| Oat | 16.00 | OM | 84.29 |
| [Barley](http://dict.cnki.net/dict_result.aspx?searchword=大麦&tjType=sentence&style=&t=barley) | 15.00 | CP | 21.82 |
| Soybean meal | 20.00 | NDF | 36.24 |
| CaHPO_4_ | 3.00 | ADF | 12.97 |
| Salt | 1.00 | Ca | 1.54 |
| Premix^(1)^ | 1.00 | P | 0.59 |
| Total | 100.00 |  |  |
| ^(1)^ vitamin A 480 IU, vitamin B_1_ 816 mg, vitamin B_2_ 333 mg, vitamin B_6_ 49 mg, vitamin D 70 U, vitamin E 21333 IU, pantothenic acid 20 mg, nicotinamide 485 mg, Cu (as copper sulphate) 11 mg, Fe (as ferrous sulphate) 35 mg, Mn (as manganese sulphate) 33 mg, Zn (as zinc sulphate) 31 mg, I (as potassium iodide) 2 mg, Se (as sodium selenite) 6 mg, and Co (as cobalt chloride) 1 mg. | | | |
