# Supplementary material for: Effect of dietary gossypol supplement on fermentation characteristics and bacterial diversity in the rumen of sheep
Source: PLoS One. 2020 Jun 10;15(6):e0234378. doi: 10.1371/journal.pone.0234378 (PMC7286523; doi:10.1371/journal.pone.0234378)
Supplement: S2 Table — (DOC) [file pone.0234378.s002.doc]

**S2 Table. Information** about the number of reads per sheep per sampling time

| **Sample name** | **Raw reads** | **Clean Reads** |
| --- | --- | --- |
| ConD20.1 | 56076 | 53271 |
| ConD20.2 | 53424 | 49866 |
| ConD20.3 | 52914 | 49871 |
| ConD20.4 | 51691 | 48202 |
| GosD20.1 | 59111 | 55578 |
| GosD20.2 | 55014 | 51906 |
| GosD20.3 | 53455 | 50619 |
| GosD20.4 | 64953 | 61203 |
| ConD47.1 | 50751 | 48400 |
| ConD47.2 | 53405 | 49805 |
| ConD47.3 | 55477 | 52493 |
| ConD47.4 | 50799 | 47382 |
| GosD47.1 | 52573 | 50142 |
| GosD47.2 | 63912 | 60992 |
| GosD47.3 | 63683 | 60148 |
| GosD47.4 | 64729 | 61227 |
| ConD20 was the samples in control group at day 20; GosD20 was the samples in gossypol group at day 20; ConD47 was the samples in control group at day 47; GosD47 was the samples in gossypol group at day 47. | | |
